# Stent versus gastrojejunostomy for the palliation of gastric outlet obstruction: a systematic review

**DOI:** 10.1186/1471-230X-7-18

**Published:** 2007-06-08

**Authors:** Suzanne M Jeurnink, Casper HJ van Eijck, Ewout W Steyerberg, Ernst J Kuipers, Peter D Siersema

**Affiliations:** 1 Department of Gastroenterology and Hepatology, Erasmus MC/University Medical Center Rotterdam, The Netherlands; 2 Department of Surgery, Erasmus MC/University Medical Center Rotterdam, The Netherlands; 3 Department of Public Health, Erasmus MC/University Medical Center Rotterdam, The Netherlands

## Abstract

**Background:**

Gastrojejunostomy (GJJ) is the most commonly used palliative treatment modality for malignant gastric outlet obstruction. Recently, stent placement has been introduced as an alternative treatment. We reviewed the available literature on stent placement and GJJ for gastric outlet obstruction, with regard to medical effects and costs.

**Methods:**

A systematic review of the literature was performed by searching PubMed for the period January 1996 and January 2006. A total of 44 publications on GJJ and stents was identified and reported results on medical effects and costs were pooled and evaluated. Results from randomized and comparative studies were used for calculating odds ratios (OR) to compare differences between the two treatment modalities.

**Results:**

In 2 randomized trials, stent placement was compared with GJJ (with 27 and 18 patients in each trial). In 6 comparative studies, stent placement was compared with GJJ. Thirty-six series evaluated either stent placement or GJJ. A total of 1046 patients received a duodenal stent and 297 patients underwent GJJ. No differences between stent placement and gastrojejunostomy were found in technical success (96% vs. 100%), early and late major complications 7% vs. 6% and 18% vs. 17%, respectively) and persisting symptoms (8% vs. 9%). Initial clinical success was higher after stent placement (89% vs. 72%). Minor complications were less frequently seen after stent placement in the patient series (9% vs. 33%), however the pooled analysis showed no differences (OR: 0.75, p = 0.8). Recurrent obstructive symptoms were more common after stent placement (18% vs. 1%). Hospital stay was prolonged after GJJ compared to stent placement (13 days vs. 7 days). The mean survival was 105 days after stent placement and 164 days after GJJ.

**Conclusion:**

These results suggest that stent placement may be associated with more favorable results in patients with a relatively short life expectancy, while GJJ is preferable in patients with a more prolonged prognosis. The paucity of evidence from large randomized trials may however have influenced the results and therefore a trial of sufficient size is needed to determine which palliative treatment modality is optimal in (sub)groups of patients with malignant gastric outlet obstruction.

## Background

Gastric Outlet Obstruction (GOO) is a common symptom in patients with cancer of the distal stomach, duodenum and pancreas. The incidence of pancreatic cancer is 7.1 per 100,000 people of which approximately 15–20% patients will develop GOO [1-4]. Other causes are periampullary carcinoma, lymphoma and metastases to the duodenum or proximal jejunum [1,3-5]. Clinical symptoms of GOO include vomiting, nausea, malnutrition and dehydration. Most patients with GOO are therefore in a poor clinical condition at presentation and have a short life expectancy if left untreated [3,6,7].

Traditionally, open gastrojejunostomy (GJJ) has been the standard palliative treatment in these patients. Open GJJ is associated with a good functional outcome and relieves symptoms in almost all patients. Nevertheless, early major complications and mortality have been reported to be substantial [3,8,9]. Most patients have delayed gastric emptying, which is defined as the inability to tolerate fluids for 8 days or more after treatment, which often causes a prolonged hospital stay [10]. In recent years, laparoscopic GJJ has been introduced as an alternative to open GJJ to relieve symptoms of malignant GOO. Laparoscopic GJJ has been reported to be less invasive and to be associated with a faster recovery compared to open GJJ, however mortality and morbidity of the procedure remain high [3,6,11,12].

Palliative stent placement for GOO was first reported in the early 1990s [13]. Stents have already extensively been used at other sites of the gastrointestinal tract, for example for palliation of dysphagia from esophageal cancer [14,15]. Stent placement for GOO has been suggested to be less invasive with a faster relief of symptoms compared to open or laparoscopic GJJ. As a consequence, hospital stay should be shorter in the majority of patients with many of them being able to eat soft solids after 1–4 days. Technical and clinical success rates have been reported to be high and mortality related to the procedure is rare after stent placement [16]. A disadvantage of stent placement is however the high rate of late major complications caused by stent migration and occlusion [1,3].

Limited data are currently available comparing stent placement and GJJ. In this study, we reviewed the available literature on stent placement and GJJ with respect to technical and clinical success, complications, hospital stay, survival and procedure-related costs.

## Methods

A systematic review of the published literature was performed by searching PubMed for the period January 1996 until December 2005, combining the following search terms: gastric outlet obstruction, duodenum, stent, gastrojejunostomy, surgical bypass and gastroenterostomy. A total of 166 studies were found using these search terms, of which 58 studies reported results on technical success, clinical success, complications, hospital stay, survival and costs for both treatment modalities. Fourteen publications were excluded for one or more of the following criteria: single case reports, abstracts, one of these treatments used in combination with a curative treatment modality or use of the same data in more than 1 article. In total, 44 studies were included (Table 1).

**Table 1:** Case series included

**Author**	**Study type**	**Years**	**Intervention**	**N**	**Age**	**Impact factor journal**
**Randomized studies**						

Mehta et al [17]	Randomized	Unknown	LGJJ	14	68	2
			Stent	13	70	
Fiori et al [18]	Randomized	2001–2002	OGJJ	9	70	1,4
			Stent	9	72	

**Comparative studies**						

Johnsson et al [19]	Prospective	1999–2004	OGJJ	15	72	2
			Stent	21	78	
Mittal et al [20]	Retrospective	1989–2002	LGJJ	14	68	5,1
			OGJJ	16	68	
			Stent	16	64	
Del Piano et al [7]	Retrospective	1997–2002	OGJJ	23	73	3,5
			Stent	24	75	
Maetani et al [8]	Retrospective	1993–2002	OGJJ	19	69	4
			Stent	20	72	
Wong et al [6]	Retrospective	1988–1998	OGJJ	17	ns	2
			Stent	6	ns	
Yim et al [21]	Retrospective	1994–1999	OGJJ	15	ns	3,5
			Stent	12	68	

**Prospective studies**						

Lillemoe et al [5]	Prospective	1994–1998	OGJJ	44	67	5,9
Van Heek et al [10]	Prospective	1998–2002	OGJJ	36	63	5,9
Jung et al [22]	Prospective	1999–2000	Stent	39	63	1,7
Pinto Pabon et al [24]	Prospective	2001	Stent	31	72	1
Kim et al [26]	Prospective	2000–2003	Stent	29	64	4
Holt et al [27]	Prospective	2000–2004	Stent	28	76	3,5
Schiefke et al [28]	Prospective	1999–2001	Stent	20	ns	3,5
Jung et al [23]	Prospective	1998–1999	Stent	19	65	5,1
Jeong et al [29]	Prospective	1999–2000	Stent	18	56	2,4
Lopera et al [30]	Prospective	2000–2001	Stent	16	58	1,7
Profili et al [31]	Prospective	1994–2000	Stent	15	65	1,2
Lee et al [32]	Prospective	1997–2000	Stent	11	68	1
Baere et al [33]	Prospective	1997	Stent	10	54	0,8
Bethge et al [34]	Prospective	1997	Stent	6	68	4,7
Espinel et al [1]	Prospective	1999–2000	Stent	6	76	1,4

**Retrospective studies**						

Brune et al [12]	Retrospective	1993–1995	LGJJ	16	67	2
Choi et al [35]	Retrospective	1999–2000	LGJJ	10	59	2
Bergamaschi et al [36]	Retrospective	1991–1996	LGJJ	9	ns	1,2
Alam et al [37]	Retrospective	1998–2000	LGJJ	8	67	2
Bergamaschi et al [36]	Retrospective	1991–1996	OGJJ	22	ns	1,2
Choi et al [35]	Retrospective	1998–2000	OGJJ	10	60	2
Telford et al [38]	Retrospective	1996–2003	Stent	176	65	3,5
Song et al [39]	Retrospective	2001–2004	Stent	102	58	1,7
Bessoud et al [11]	Retrospective	Unknown	Stent	72	62	1,7
Nassif et al [40]	Retrospective	1998–2001	Stent	63	73	4
Kim et al [26]	Retrospective	1995–1999	Stent	49	57	0,2
Adler et al [4]	Retrospective	1998–2001	Stent	36	61	4,7
Kaw et al [41]	Retrospective	1998–2001	Stent	33	62	2
Razzaq et al [9]	Retrospective	1996–2000	Stent	28	69	0,9
Park et al [42]	Retrospective	1996–1999	Stent	24	43	5,1
Aviv et al [43]	Retrospective	1998–1999	Stent	15	61	1,5
Feretis et al [44]	Retrospective	1993–1994	Stent	12	64	4
Soetikno et al [46]	Retrospective	1995–1997	Stent	12	60	3,5
Yates et al [47]	Retrospective	1994–1996	Stent	11	71	4
Feretis et al [45]	Retrospective	Unknown	Stent	10	72	3,5
Nevitt et al [48]	Retrospective	1991–1997	Stent	8	63	2
Venu et al [49]	Retrospective	Unknown	Stent	8	66	4
Ely et al [50]	Retrospective	1998–2002	Stent	5	65	2

ns = not specified

OGJJ = Open gastrojejunostomy

LGJJ = Laparoscopic gastrojejunostomy

### Definitions

For this review, we used the following definitions:

- Technical success: Adequate positioning and deployment of the stent or technical feasibility to perform a GJJ.

- Clinical success: Relief of symptoms and/or improvement of oral intake.

- Major complications: Life-threatening or severe complications such as perforation, stent migration, hemorrhage, fever, jaundice or severe pain, often requiring additional treatment and hospitalization. Major complications were divided in early (≤ 7 days after treatment) or late (>7 days after treatment) complications.

- Minor complications: Complications which were not life-threatening or moderately severe, such as mild pain, wound infection, mild fever or occasionally vomiting without obstruction.

- Persistent obstruction: Persistence of obstructive symptoms after the intervention.

- Recurrent obstruction: Recurrent obstructive symptoms during follow-up.

### Statistics

Technical and clinical success, complications, persistent and recurrent obstruction, hospital stay and survival rates were pooled. Odds ratios (OR) with 95% confidence interval (CI) were calculated for technical success, clinical success, early and late major complications, and minor complications using data from the randomized and comparative studies. Odds ratios were not calculated for a study when the event was detected in all patients. Calculations were done with SPSS 12.0 and RevMan 4.2. A p < 0.05 was considered to be statistically significant.

## Results

### Study characteristics

A total of 44 studies were included in this review [1,4-12,17-50]. Study characteristics are shown in Table 1. Only two studies had a randomized design (with 27 and 18 patients included[17,18]). Stent placement was prospectively or retrospectively compared with GJJ in 6 studies [6-8,19-21]. Two retrospective studies compared laparoscopic GJJ with open GJJ [35,36]. Thirty-four studies evaluated either stent placement or GJJ. According to the Delphi criteria, we assessed the quality of the randomized and comparative trials with regard to: a) method of randomization, b) treatment allocation, c) similarity between groups, d) specification of eligible criteria, e) blinded outcome of assessor, care provider and patient, f) information on primary outcome, g) and intention-to-treat analysis [51]. Applying these criteria made clear that the quality of the trials was limited.

### Patient characteristics

A total of 1046 patients received a duodenal stent (mean age: 64 years) and 297 patients underwent GJJ (mean age: 67 years).

Biliary drainage some time before stent placement was performed in 76/579 (13%) patients, during stent placement in 34/579 (6%), and after stent placement in 31/579 (5%) [1,4,8,11,17,19,20,30,38-40,43,46]. A biliary drainage procedure some time before GJJ was performed in 18/102 (18%) patients, during GJJ in 16/102 (16%) and after GJJ in 17/102 (17%) [8,12,17,19,20,20,37].  Results on study outcomes are shown in Table 2.

### Technical success

Stent placement was usually performed by endoscopy in combination with fluoroscopy. The stents that were used included enteral Wallstents and Niti-S stents, esophageal Memotherm stents, Ultraflex stents, Choo stents, Gianturco-Z stents, Song stent, Flamingo Wallstents and Endocoil stents. The surgical technique that was used for the GJJ included an open or laparoscopic procedure that was performed in an antecolic or retrocolic way.

Stent placement was technically feasible in 972/1012 (96%) patients and GJJ in 203/204 (99%) patients (Table 3). The main reasons for technical failure of stent placement were dislocation of the stent during the procedure, no passage of the guidewire through the stricture, failure to deploy or release the stent from the delivery system. The reason for technical failure to perform a GJJ was the finding of peritoneal carcinomatosis during the procedure.

**Table 3:** Summary of the main study outcomes of stent placement and gastrojejunostomy in patients with malignant gastric outlet obstruction

	**Stent**	**GJJ**
Technical success (%)	972/1012 (96)	203/204 (99)
Clinical success (%)	890/1000 (89)	79/110 (72)
Complications (%)		
Early major complications	43/609 (7)	6/159 (4)
Late major complications	171/950 (18)	34/201 (17)
Minor complications	66/732 (9)	66/201 (33)
Persistent obstructive symptoms	43/535 (8)	10/106 (9)
Reintervention	147/814 (18)	1/138 (1)
Mean hospital stay (days, [range])	7 (2–18)	13 (7–30)
Mean survival (days, [range])	105 (23–210)	164 (64–348)

### Clinical success

Clinical success was 89% (890/1000 patients) after stent placement and 72% (79/110) after GJJ (Table 3). Information on food intake was available in most studies evaluating stent placement and in only one study that had included a small number of patients receiving a GJJ [19]. Based on the available data, we scored the results on food intake using the standardized Gastric Outlet Obstruction Scoring System (GOOSS) score, with 0 = no oral intake, 1 = liquids only, 2 = soft foods and 3 = solid food/full diet [4]. Food intake before the intervention was poor with no difference between patients undergoing stent placement or GJJ. The mean GOOSS score was 0 in 148/238 (62%) patients, 1 in 78/238 (33%) and 2 in 12/238 (5%). Following treatment with a stent or GJJ, food intake improved in the majority of patients. After stent placement, the GOOSS score was 0 in 18/306 (6%) patients, 1 in 68/306 (22%), 2 in 122/306 (40%) and 3 in 98/306 (32%). One week after GJJ, the GOOSS score was 0 in 5/14 (36%) patients, 1 in 7/14 (50%), 2 in 1/14 (7%) and 3 in 1/14 (7%).

### Complications

Early major complications were not different between stent placement (7%; 43/609) and GJJ (4%; 6/159)(Table 3). Early major complications after stent placement were mainly stent migration and dysfunction of the stent and after GJJ, jaundice and bleeding. In most patients with early major complications, a reintervention was performed. In addition, no differences in late major complications between stent placement (18%; 171/950) and GJJ (17%; 34/201) were found. The most commonly observed late complications after stent placement were stent migration and occlusion either by tumor in- or overgrowth or food. After GJJ, late major complications included leakage at the anastomotic site, fever and dysfunction of the GJJ.

Minor complications occurred more frequently after GJJ (33%; 66/201) than after stent placement (9%; 66/732). Minor complications after stent placement included mild pain in the upper abdominal region, vomiting or mild bleeding, whereas after GJJ delayed gastric emptying and wound infections were most frequently seen.

Persistent obstructive symptoms after treatment occurred in 43/535 (8%) patients after stent placement and in 10/106 (9%) following GJJ.

A reintervention for recurrent obstructive symptoms was more frequently performed after stent placement than after GJJ (18%; 147/814 vs. 1%; 1/138). Causes of recurrent obstruction after stent placement included stent occlusion by tumor in- and overgrowth or food.

### Hospital stay and survival

Mean hospital stay was shorter after stent placement (7 days, n = 324) than after GJJ (13 days, n = 385). Mean survival after stent placement was 105 days (n = 923) and after GJJ 164 days (n = 246).

### Costs

Total costs of stent placement and GJJ were compared in three non-randomized studies [19-21]. In the study by Yim et al [21], mean total costs were $9,921 for stent placement and $28,173 for OGJJ. Only procedural costs were used in this calculation. In the study by Mittal et al [20], information was collected on procedural and post procedural costs. Mean costs were $8,680 for stent placement, $20,060 for OGJJ and $16,552 for LGJJ. Johnsson et al [19] included procedural costs, postoperative care, hospital stay and additional procedures. Mean costs were $8,163 for stent placement and $10,224 for OGJJ.

### Odds ratios for available comparative and randomized studies

Odds ratios were analyzed for technical success, clinical success, early major complications, late major complications and minor complications using the two randomized studies [17,18] and 6 comparative studies [6-8,19-21]. The results showed no difference in technical success rate between stent placement and GJJ (OR: 0.22, CI: 0.02–2.1, p = 0.2). The clinical success rate seemed however higher after stent placement than after GJJ (OR: 3.39, CI: 0.8–14.3, p = 0.1). The results for early major and late major complications showed no clear differences between stent placement and GJJ (OR: 0.49, CI: 0.1–2.6, p = 0.4 and OR: 0.74, CI: 0.1–4.0, p = 0.7, respectively). Finally, no differences in minor complications between the two treatment modalities were found (OR: 0.75, CI: 0.1–5.0, p = 0.8).

## Discussion

This review summarizes the published results on duodenal stent placement and GJJ as palliative treatment modalities for GOO. There is a paucity of evidence to conclude that either one of these two treatment modalities gave better treatment results. The results of this review suggest however that patients with a duodenal stent have a shorter hospital stay, a more frequent and faster relief of obstructive symptoms, which may be associated with fewer minor complications than those treated with a GJJ. Nevertheless, patients after a GJJ have fewer recurrences of obstructive symptoms and therefore the need for reinterventions is lower in GJJ patients than in those being treated with a stent.

The main objective of a palliative procedure in patients with malignant GOO is to restore the ability to eat. This review demonstrates that clinical success, defined as improvement of food intake and/or relief of symptoms, was more common after stent placement than after GJJ, with the OR also showing better, but statistically not significant, results after stent placement than after GJJ (OR = 3.39, CI: 0.8–14.3, p = 0.1). As stent placement is a less invasive treatment than GJJ, this may well explain why a faster relief of symptoms is seen with this treatment modality. In addition, the position of the anastomosis at the greater curvature after a GJJ may also contribute to the less favorable results following a surgical procedure. Nevertheless, our results are only based on studies with small patient numbers, and more and larger randomized studies are needed.

This review showed no differences in early and late major complications between stent placement and GJJ, which was confirmed by the ORs obtained from the randomized and comparative studies. Minor complications occurred more frequently after GJJ than after stent placement if all studies were compared. The OR however, did not indicate a difference between stent placement and GJJ (OR: 0.75, CI: 0.11–5.04, p = 0.77). Remarkably, complication rates varied widely in the reviewed studies, which may have been caused by differences in patient age, clinical condition, sample size, operator experience and in the definitions used for complications in the different series and studies that were reviewed. In addition, it was not always possible to detect whether a complication was indeed associated with the treatment modality or with progression of the malignant disorder.

Recurrent obstructive symptoms, necessitating a reintervention, occurred more frequently after stent placement than after GJJ. The majority of recurrent obstructive symptoms after stent placement were caused by stent occlusion from either tumor in- or overgrowth, or food obstruction. Duodenal stent obstruction by tumor in- or overgrowth remains a problem, especially when non-covered stents are used. The use of covered stents in the duodenum may however lead to a higher incidence of stent migration and may also lead to an increased incidence of biliary obstruction and even pancreatitis due to obstruction of the common bile duct and/or pancreatic duct by the covered device [23,25,29,30,42]. Stent migration seems to occur in a shorter time period (range: 1–121 days) after stent placement than recurrent obstructive symptoms caused by tumor in- or overgrowth or food debris (range: 11–273 days). In addition, stent migration seems to occur at a shorter time period and more frequently after placement of a covered stent (19%) than after placement of a uncovered stent (6%) [11,23,24,26,29-31,39,42,47].

Our review suggests that initial costs are lower for stent placement than for a surgical procedure. However, in the few studies that evaluated costs, reintervention and additional care costs were not taken into consideration [19-21]. As GJJ was found to be associated with a prolonged hospital stay, initial costs are likely to be higher following GJJ. Following stent placement however, a higher incidence of reinterventions for recurrent obstruction is likely to occur and this may result in more or less similar costs for GJJ and stent placement on the long term. A future cost-analysis study is needed that includes all costs of stent placement and GJJ involved in the whole period of time that these patients survive.

A number of issues are important to consider before concluding that either one of these treatment modalities is favorable in patients with a GOO. First, only 2 randomized trials and 6 comparative studies have so far been performed including small patient numbers. The prospective and retrospective design of most studies included in this review resulted in a minimal access to primary study outcomes and a comparison between potentially noncomparable patient populations. In most studies, no differentiation was made with respect to underlying malignancies. It is well known that survival in patients with GOO caused by pancreatic carcinoma is shorter than that in patients with gastric- or duodenal carcinoma [52]. Pancreatic cancer was the most common cause of GOO in various series. However, specific results for different types of patients were not available. Therefore survival rates may have been over- or underestimated depending on the type of patients that were included.

Secondly, several stent types were used in the different studies, whereas in some studies also more than one stent type was used. Again, specific data on outcome for individual stent types were often not available. Moreover, in several studies, esophageal stents rather than enteral stents were used. This could have influenced the complication rate, as esophageal stents are often covered, in contrast to enteral stents, resulting in an increased risk of stent migration [53]. Moreover, as esophageal stents, in contrast to enteral stents, cannot be placed through-the-scope, placement of these devices may have been technically more demanding. Only two studies compared open GJJ with laparoscopic GJJ. These comparative studies suggested that both hospital stay and time to restore the ability to eat were shorter after laparoscopic GJJ than after open GJJ. However additional, and preferably randomized studies are needed before a recommendation in favor of a laparoscopic procedure can be given in these patients.

Finally, publication bias (the selective reporting of studies with positive results) may result in overestimation of technical and clinical success rates and survival, and underestimation of complications and hospital stay. We assessed publication bias and found no clear effect of sample size or impact factor of the journal on the different endpoints (results not shown). Using the Delphi criteria to assess the quality of the randomized and comparative trials, made clear that the quality of the assessed trials was limited [51]. In addition, the quality of the patient series was low because of small patient populations and minimal access to primary data. A high-quality trial may alter the interpretation of the benefit of the two treatment modalities. The results of this review should not be considered as a critical appraisal, but addresses the possible differences in treatment effects between stent placement and GJJ.

## Conclusion

Despite the above-mentioned limitations, it seems reasonable to suggest that stent placement is associated with more favorable short-term results, whereas GJJ may be a better treatment option in patients with a more prolonged survival. The results of this review suggest that a trial with a sufficient number of patients is indicated in which patients with malignant GOO are randomized to stent placement or GJJ in order to define treatment guidelines for individual patients based on the underlying disorder and prognosis. In addition, a longer follow-up of patients is needed to assess the different endpoints, and, if indicated, to perform a cost-effectiveness analysis.

## Competing interests

The author(s) declare that they have no competing interests.

## Authors' contributions

S.J. performed a systematic search, performed the statistical analyses and drafted the manuscript, C.E. helped to draft the manuscript, E.S. helped with the statistical analyses and helped to draft the manuscript. E.K. helped to draft the manuscript, P.S. helped with the systematic search and helped to draft the manuscript. All authors read and approved the final manuscript.

**Table 2:** Results on technical and clinical success, hospital stay, complications, survival and 30-day mortality

**Author**	**Intervention**	**N**	**Technical success **	**Clinical success **	**Hospital stay **	**Major complications **		**Minor complications **	**Survival **	**30-day mortality **
			**(%)**	**(%)**	**(days)**	**Early (%) **	**Late (%)**	**(%)**	**(days)**	**(%)**
**Randomized studies**										

Mehta et al	LGJJ	14	93	ns	11	0	0	62	ns	23
	Stent	13	77	ns	5	0	0	0	ns	20
Fiori et al	OGJJ	9	100	89	10	11	0	11	ns	ns
	Stent	9	100	100	3,1	11	0	11	ns	ns

**Comparative studies**										

Johnsson et al	OGJJ	15	100	81	15	0	13	ns	99	27
	Stent	21	100	100	7	5	14	5	76	29
Mittal et al	LGJJ	14	ns	ns	7	0	36	7	119	ns
	OGJJ	16	ns	ns	10	0	31	ns	120	ns
	Stent	16	ns	ns	2	0	0	0	56	ns
Del Piano et al	OGJJ	23	100	56	24	0	30	61	70	30
	Stent	24	96	92	3	0	17	ns	96	0
Maetani et al	OGJJ	19	100	84	30	26	0	5	79	16
	Stent	20	100	80	15	5	25	10	55	25
Wong et al	OGJJ	17	ns	ns	15	ns	ns	ns	64	18
	Stent	6	ns	ns	4	ns	ns	ns	98	0
Yim et al	OGJJ	15	ns	ns	14	ns	ns	ns	92	ns
	Stent	12	94	81	4	8	17	ns	94	ns

**Prospective studies**										

Lillemoe et al	OGJJ	44	100	ns	8,5	0	0	32	249	0
Van Heek et al	OGJJ	36	100	ns	11	0	21	25	216	3
Jung et al	Stent	39	97	95	ns	8	28	3	134	10
Pinto Pabon et al	Stent	31	100	90	ns	0	10	29	92	29
Kim et al	Stent	29	90	96	18	0	29	ns	124	0
Holt et al	Stent	28	93	93	7	0	21	ns	51	42
Schiefke et al	Stent	20	100	100	ns	ns	ns	ns	144	ns
Jung et al	Stent	19	95	100	ns	26	0	ns	ns	0
Jeong et al	Stent	18	100	94	ns	6	22	11	85	ns
Lopera et al	Stent	16	94	81	ns	19	0	13	84	ns
Profili et al	Stent	15	100	93	ns	0	14	14	ns	0
Lee et al	Stent	11	87	82	ns	0	0	64	ns	ns
Baere de et al	Stent	10	100	80	2	10	10	20	93	ns
Bethge et al	Stent	6	100	100	ns	0	33	0	23	83
Espinel et al	Stent	6	100	100	2,5	0	0	ns	98	0

**Retrospective studies**										

Brune et al	LGJJ	16	100	81	7	6	0	19	87	0
Choi et al	LGJJ	10	100	100	9	0	0	30	ns	ns
Bergamaschi et al	LGJJ	9			10	ns	ns	ns	348	ns
Alam et al	LGJJ	8	100	88	7	13	75	ns	ns	ns
Bergamaschi et al	OGJJ	22			15	ns	ns	294	ns	ns
Choi et al	OGJJ	10	100	100	13	0	10	70	ns	ns
Telford et al	Stent	176	97	84	ns	ns	9	6	97	ns
Song et al	Stent	102	99	84	ns	ns	9	2	92	2
Bessoud et al	Stent	72	97	90	ns	1	14	1	120	ns
Nassif et al	Stent	63	95	92	6	33	67	ns	210	ns
Kim et al	Stent	49	100	92	7	17	10	70	18	ns
Adler et al	Stent	36	100	97	ns	3	3	22	83	ns
Kaw et al	Stent	33	97	88	ns	0	13	12	102	ns
Razzaq et al	Stent	28	96	91	ns	ns	27	4	95	18
Park	Stent	24	75	67	ns	4	38	ns	129	0
Aviv et al	Stent	15	93	93	ns	13	20	ns	72	ns
Feretis et al	Stent	12	100	92	ns	8	0	0	ns	0
Soetikno et al	Stent	12	100	75	2	ns	ns	25	ns	ns
Yates	Stent	11	91	91	ns	ns	ns	63	77	ns
Feretis et al	Stent	10	100	100	ns	0	20	ns	ns	ns
Nevitt et al	Stent	8	100	88	ns	0	38	ns	141	0
Venu et al	Stent	8	100	100	ns	0	10	0	ns	13
Ely et al	Stent	5	100	100	ns	0	0	20	ns	ns

ns = not specified

OGJJ = Open gastrojejunostomy

LGJJ = Laparoscopic gastrojejunostomy

## Pre-publication history

The pre-publication history for this paper can be accessed here:



## References

[B1] Espinel J, Vivas S, Munoz F, Jorquera F, Olcoz JL (2001). Palliative treatment of malignant obstruction of gastric outlet using an endoscopically placed enteral Wallstent. Dig Dis Sci.

[B2] (2006). Kennisnetwerk Integrale kankercentra. http://www.ikc.nl.

[B3] Lopera JE, Brazzini A, Gonzales A, Castaneda-Zuniga WR (2004). Gastroduodenal stent placement: current status. Radiographics.

[B4] Adler DG, Baron TH (2002). Endoscopic palliation of malignant gastric outlet obstruction using self-expanding metal stents: experience in 36 patients. Am J Gastroenterol.

[B5] Lillemoe KD, Cameron JL, Hardacre JM, Sohn TA, Sauter PK, Coleman J, Pitt HA, Yeo CJ (1999). Is prophylactic gastrojejunostomy indicated for unresectable periampullary cancer? A prospective randomized trial. Ann Surg.

[B6] Wong YT, Brams DM, Munson L, Sanders L, Heiss F, Chase M, Birkett DH (2002). Gastric outlet obstruction secondary to pancreatic cancer: surgical vs endoscopic palliation. Surg Endosc.

[B7] Del Piano M, Ballare M, Montino F, Todesco A, Orsello M, Magnani C, Garello E (2005). Endoscopy or surgery for malignant GI outlet obstruction?. Gastrointest Endosc.

[B8] Maetani I, Tada T, Ukita T, Inoue H, Sakai Y, Nagao J (2004). Comparison of duodenal stent placement with surgical gastrojejunostomy for palliation in patients with duodenal obstructions caused by pancreaticobiliary malignancies. Endoscopy.

[B9] Razzaq R, Laasch HU, England R, Marriott A, Martin D (2001). Expandable metal stents for the palliation of malignant gastroduodenal obstruction. Cardiovasc Intervent Radiol.

[B10] Van Heek NT, De Castro SM, van Eijck CH, van Geenen RC, Hesselink EJ, Breslau PJ, Tran TC, Kazemier G, Visser MR, Busch OR, Obertop H, Gouma DJ (2003). The need for a prophylactic gastrojejunostomy for unresectable periampullary cancer: a prospective randomized multicenter trial with special focus on assessment of quality of life. Ann Surg.

[B11] Bessoud B, T. B, Denys A, Kuoch V, Ducreux M, Precetti S, Roche A, Menu Y (2005). Malignant gastroduodenal obstruction: palliation with self-expanding metallic stents. J Vasc Interv Radiol.

[B12] Brune IB, Feussner H, Neuhaus H, Classen M, Siewert JR (1997). Laparoscopic gastrojejunostomy and endoscopic biliary stent placement for palliation of incurable gastric outlet obstruction with cholestasis. Surg Endosc.

[B13] Kozarek RA, Ball TJ, Patterson DJ (1992). Metallic self-expanding stent application in the upper gastrointestinal tract: caveats and concerns. Gastrointest Endosc.

[B14] Homs MY, Essink-Bot ML, Borsboom GJ, Steyerberg EW, Siersema PD (2004). Quality of life after palliative treatment for oesophageal carcinoma -- a prospective comparison between stent placement and single dose brachytherapy. Eur J Cancer.

[B15] Homs MY, Steyerberg EW, Eijkenboom WM, Tilanus HW, Stalpers LJ, Bartelsman JF, van Lanschot JJ, Wijrdeman HK, Mulder CJ, Reinders JG, Boot H, Aleman BM, Kuipers EJ, Siersema PD (2004). Single-dose brachytherapy versus metal stent placement for the palliation of dysphagia from oesophageal cancer: multicentre randomised trial. Lancet.

[B16] Baron TH, Schofl R, Puespoek A, Sakai Y (2001). Expandable metal stent placement for gastric outlet obstruction. Endoscopy.

[B17] Mehta S, Hindmarsh A, Cheong E, Cockburn J, Saada J, Tighe R, Lewis MP, Rhodes M (2006). Prospective randomized trial of laparoscopic gastrojejunostomy versus duodenal stenting for malignant gastric outflow obstruction. Surg Endosc.

[B18] Fiori E, Lamazza A, Volpino P, Burza A, Paparelli C, Cavallaro G, Schillaci A, Cangemi V (2004). Palliative management of malignant antro-pyloric strictures. Gastroenterostomy vs. endoscopic stenting. A randomized prospective trial. Anticancer Res.

[B19] Johnsson E, Thune A, Liedman B (2004). Palliation of malignant gastroduodenal obstruction with open surgical bypass or endoscopic stenting: clinical outcome and health economic evaluation. World J Surg.

[B20] Mittal A, Windsor J, Woodfield J, Casey P, Lane M (2004). Matched study of three methods for palliation of malignant pyloroduodenal obstruction. Br J Surg.

[B21] Yim HB, Jacobson BC, Saltzman JR, Johannes RS, Bounds BC, Lee JH, Shields SJ, Ruymann FW, Van DJ, Carr-Locke DL (2001). Clinical outcome of the use of enteral stents for palliation of patients with malignant upper GI obstruction. Gastrointest Endosc.

[B22] Jung GS, Song HY, Seo TS, Park SJ, Koo JY, Huh JD, Cho YD (2002). Malignant gastric outlet obstructions: treatment by means of coaxial placement of uncovered and covered expandable nitinol stents. J Vasc Interv Radiol.

[B23] Jung GS, Song HY, Kang SG, Huh JD, Park SJ, Koo JY, Cho YD (2000). Malignant gastroduodenal obstructions: treatment by means of a covered expandable metallic stent-initial experience. Radiology.

[B24] Pinto Pabon I, Diaz LP, Ruiz De Adana JC, Lopez HJ (2001). Gastric and duodenal stents: follow-up and complications. Cardiovasc Intervent Radiol.

[B25] Kim GH, Kang DH, Lee DH, Heo J, Song GA, Cho M, Yang US (2004). Which types of stent, uncovered or covered, should be used in gastric outlet obstructions?. Scand J Gastroenterol.

[B26] Kim JH, Yoo BM, Lee KJ, Hahm KB, Cho SW, Park JJ, Kim SS, Park HC, Kim JH (2001). Self-expanding coil stent with a long delivery system for palliation of unresectable malignant gastric outlet obstruction: a prospective study. Endoscopy.

[B27] Holt AP, Patel M, Ahmed MM (2004). Palliation of patients with malignant gastroduodenal obstruction with self-expanding metallic stents: the treatment of choice?. Gastrointest Endosc.

[B28] Schiefke I, Zabel-Langhennig A, Wiedmann M, Huster D, Witzigmann H, Mossner J, Berr F, Caca K (2003). Self-expandable metallic stents for malignant duodenal obstruction caused by biliary tract cancer. Gastrointest Endosc.

[B29] Jeong JY, Han JK, Kim AY, Lee KH, Lee JY, Kang JW, Kim TJ, Shin SH, Choi BI (2002). Fluoroscopically guided placement of a covered self-expandable metallic stent for malignant antroduodenal obstructions: preliminary results in 18 patients. AJR Am J Roentgenol.

[B30] Lopera JE, Alvarez O, Castano R, Castaneda-Zuniga W (2001). Initial experience with Song's covered duodenal stent in the treatment of malignant gastroduodenal obstruction. J Vasc Interv Radiol.

[B31] Profili S, Meloni GB, Bifulco V, Conti M, Feo CF, Canalis GC (2001). Self-expandable metal stents in the treatment of antro-pyloric and/or duodenal strictures. Acta Radiol.

[B32] Lee JM, Han YM, Lee SY, Kim CS, Yang DH, Lee SO (2001). Palliation of postoperative gastrointestinal anastomotic malignant strictures with flexible covered metallic stents: preliminary results. Cardiovasc Intervent Radiol.

[B33] de Baere T, Harry G, Ducreux M, Elias D, Briquet R, Kuoch V, Roche A (1997). Self-expanding metallic stents as palliative treatment of malignant gastroduodenal stenosis. AJR Am J Roentgenol.

[B34] Bethge N, Breitkreutz C, Vakil N (1998). Metal stents for the palliation of inoperable upper gastrointestinal stenoses. Am J Gastroenterol.

[B35] Choi YB (2002). Laparoscopic gatrojejunostomy for palliation of gastric outlet obstruction in unresectable gastric cancer. Surg Endosc.

[B36] Bergamaschi R, Marvik R, Thoresen JE, Ystgaard B, Johnsen G, Myrvold HE (1998). Open versus laparoscopic gastrojejunostomy for palliation in advanced pancreatic cancer. Surg Laparosc Endosc.

[B37] Alam TA, Baines M, Parker MC (2003). The management of gastric outlet obstruction secondary to inoperable cancer. Surg Endosc.

[B38] Telford JJ, Carr-Locke DL, Baron TH, Tringali A, Parsons WG, Gabbrielli A, Costamagna G (2004). Palliation of patients with malignant gastric outlet obstruction with the enteral Wallstent: outcomes from a multicenter study. Gastrointest Endosc.

[B39] Song HY, Shin JH, Yoon CJ, Lee GH, Kim TW, Lee SK, Yook JH, Kim BS (2004). A dual expandable nitinol stent: experience in 102 patients with malignant gastroduodenal strictures. J Vasc Interv Radiol.

[B40] Nassif T, Prat F, Meduri B, Fritsch J, Choury AD, Dumont JL, Auroux J, Desaint B, Boboc B, Ponsot P, Cervoni JP (2003). Endoscopic palliation of malignant gastric outlet obstruction using self-expandable metallic stents: results of a multicenter study. Endoscopy.

[B41] Kaw M, Singh S, Gagneja H, Azad P (2003). Role of self-expandable metal stents in the palliation of malignant duodenal obstruction. Surg Endosc.

[B42] Park KB, Do YS, Kang WK, Choo SW, Han YH, Suh SW, Lee SJ, Park KS, Choo IW (2001). Malignant obstruction of gastric outlet and duodenum: palliation with flexible covered metallic stents. Radiology.

[B43] Aviv RI, Shyamalan G, Khan FH, Watkinson AF, Tibballs J, Caplin M, Winslett M (2002). Use of stents in the palliative treatment of malignant gastric outlet and duodenal obstruction. Clin Radiol.

[B44] Feretis C, Benakis P, Dimopoulos C, Manouras A, Tsimbloulis B, Apostolidis N (1997). Duodenal obstruction caused by pancreatic head carcinoma: palliation with self-expandable endoprostheses. Gastrointest Endosc.

[B45] Feretis C, Benakis P, Dimopoulos C, Georgopoulos K, Milas F, Manouras A, Apostolidis N (1996). Palliation of malignant gastric outlet obstruction with self-expanding metal stents. Endoscopy.

[B46] Soetikno RM, Lichtenstein DR, Vandervoort J, Wong RC, Roston AD, Slivka A, Montes H, Carr-Locke DL (1998). Palliation of malignant gastric outlet obstruction using an endoscopically placed Wallstent. Gastrointest Endosc.

[B47] Yates MR, Morgan DE, Baron TH (1998). Palliation of malignant gastric and small intestinal strictures with self-expandable metal stents. Endoscopy.

[B48] Nevitt AW, Vida F, Kozarek RA, Traverso LW, Raltz SL (1998). Expandable metallic prostheses for malignant obstructions of gastric outlet and proximal small bowel. Gastrointest Endosc.

[B49] Venu RP, Pastika BJ, Kini M, Chua D, Christian R, Schlais J, Brown RD (1998). Self-expandable metal stents for malignant gastric outlet obstruction: a modified technique. Endoscopy.

[B50] Ely CA, Arregui ME (2003). The use of enteral stents in colonic and gastric outlet obstruction. Surg Endosc.

[B51] Verhagen AP, de Vet HC, de Bie RA, Kessels AG, Boers M, Bouter LM, Knipschild PG (1998). The Delphi list: a criteria list for quality assessment of randomized clinical trials for conducting systematic reviews developed by Delphi consensus. J Clin Epidemiol.

[B52] Jemal A, Siegel R, Ward E, Murray T, Xu J, Smigal C, Thun MJ (2006). Cancer statistics, 2006. CA Cancer J Clin.

[B53] Verschuur EM, Homs MY, Steyerberg EW, Haringsma J, Wahab PJ, Kuipers EJ, Siersema PD (2006). A new esophageal stent design (Niti-S stent) for the prevention of migration: a prospective study in 42 patients. Gastrointest Endosc.

